# The association between red blood cell distribution width-to-albumin ratio and chest pain prevalence in US adults: A cross-sectional analysis of NHANES 2005–2018

**DOI:** 10.1097/MD.0000000000043200

**Published:** 2025-07-04

**Authors:** Minmin Zhu, Dongxiao Huang, Hao Xi

**Affiliations:** a Department of Anesthesiology, Wuxi No. 2 People’s Hospital, Wuxi, Jiangsu Province, China.

**Keywords:** chest pain, cross-sectional study, NHANES, RAR

## Abstract

The red blood cell distribution width-to-albumin ratio (RAR), a novel marker reflecting both inflammation and nutritional status, has emerged as a potential clinical biomarker. However, its link to chest pain in general populations remains underexplored. This study is the first to assess the association between RAR and chest pain prevalence using data from the nationally representative National Health and Nutrition Examination Survey cohort. Data from 21,174 adults in the 2005–2018 National Health and Nutrition Examination Survey were analyzed. Chest pain was assessed via the Rose Angina Questionnaire. RAR was calculated by dividing red cell distribution width by serum albumin. Multivariable logistic regression models incorporating survey weights, restricted cubic spline analyses, and subgroup analyses were used to evaluate the association, adjusting for demographic, socioeconomic, lifestyle, and clinical factors. A nonlinear relationship was found between RAR and chest pain, with an inflection point at RAR = 3.59. Below this point, each unit increase in RAR was associated with a 32% higher likelihood of chest pain (odds ratio = 1.32, 95% confidence interval: 1.17–1.49, *P* < .0001). Above 3.59, the association weakened. Compared to the lowest quartile (Q1), individuals in the highest RAR quartile (Q4) had a 26% increased prevalence of chest pain (odds ratio = 1.26, 95% confidence interval 1.15–1.39, *P* < .0001). Subgroup analysis showed stronger associations in smokers (interaction *P* = .016), with consistent results across age and racial/ethnic groups. Higher RAR is independently associated with increased chest pain prevalence, especially below 3.59, suggesting its value in risk stratification, particularly among smokers.

## 1. Introduction

Chest pain is a common clinical symptom with a broad differential diagnosis, encompassing cardiovascular, respiratory, gastrointestinal, and musculoskeletal causes.^[1]^ Among these, acute coronary syndromes (ACS) are of particular concern due to their high mortality risk.^[2]^ In the United States, chest pain is the second most frequent reason for emergency department visits, leading to millions of hospital admissions each year for diagnostic evaluation.^[3]^ Its lifetime prevalence is estimated at 20% to 40%, reflecting a significant burden on both clinical practice and public health systems.^[4]^ Although many cases are attributed to nonserious conditions, a substantial proportion are linked to cardiovascular diseases, particularly coronary artery disease, which pose serious health threats. cardiovascular diseases remains the leading cause of death in the US, affecting more than 18.2 million adults.^[5]^ Despite improvements in diagnostic tools, risk assessment, and therapeutic approaches, early recognition and prevention of high-risk cases continue to present major clinical challenges.

Emerging evidence indicates that inflammation plays a pivotal role in the pathogenesis of chest pain, particularly of cardiovascular origin.^[6]^ Systemic inflammatory processes contribute to endothelial dysfunction and destabilization of atherosclerotic plaques, thereby increasing the likelihood of ischemic events.^[7]^ Elevated levels of inflammatory biomarkers such as C-reactive protein and interleukin-6 (IL-6) have been linked to heightened risk of ACS, supporting the association between inflammation and the severity of chest pain.^[8]^ In parallel, malnutrition – characterized by low serum albumin or micronutrient deficiencies – may exacerbate inflammation and oxidative stress, compounding cardiovascular risk.^[9]^ These findings underscore a complex interplay between immune dysregulation, metabolic imbalance, and the clinical manifestation of chest pain, suggesting that targeting inflammatory pathways could enhance diagnostic precision and therapeutic strategies.

Red blood cell distribution width-to-albumin ratio (RAR) has recently emerged as a promising composite biomarker reflecting systemic inflammation, oxidative stress, and nutritional status.^[10]^ Red cell distribution width (RDW) is a standard hematological parameter that reflects erythrocyte size variability and has gained recognition not only in diagnosing anemia but also as a prognostic indicator in cardiovascular, cerebrovascular, and metabolic disorders.^[11]^ Serum albumin is the most abundant circulating protein and serves as an indicator of nutritional and physiological resilience due to its anti-inflammatory, antioxidant, and vascular-protective properties.^[12]^ Combined as RAR, these 2 measures may better capture the cumulative burden of chronic inflammation and metabolic dysfunction than either marker alone.^[13]^ Prior studies have shown RAR’s predictive superiority in various clinical settings, including ACS, heart failure, stroke, and chronic kidney disease, suggesting it reflects systemic vulnerability and biological aging.^[14]^ However, its association with more prevalent yet clinically significant symptoms such as chest pain has not been thoroughly investigated.

This study is the first to utilize nationally representative data from the National Health and Nutrition Examination Survey (NHANES) to investigate the association between RAR and chest pain prevalence in the general adult population. By examining this novel biomarker within a broad epidemiological context, we aim to address a key knowledge gap and explore the potential of RAR in enhancing symptom-based risk stratification in clinical practice.

## 2. Materials and methods

### 2.1. Study population

This cross-sectional analysis was based on data from NHANES, a continuous program conducted by the Centers for Disease Control and Prevention to assess the health and nutritional status of the US civilian, noninstitutionalized population. NHANES utilizes a stratified, multistage probability sampling strategy to ensure nationally representative estimates. Data are collected via in-home interviews, standardized physical examinations at mobile examination center, and laboratory assessments.^[15]^ All NHANES protocols were approved by the National Center for Health Statistics Research Ethics Review Board, and written informed consent was obtained from all participants. Detailed survey procedures are publicly available on the NHANES website (https://www.cdc.gov/nchs/nhanes/).

**Figure 1.**
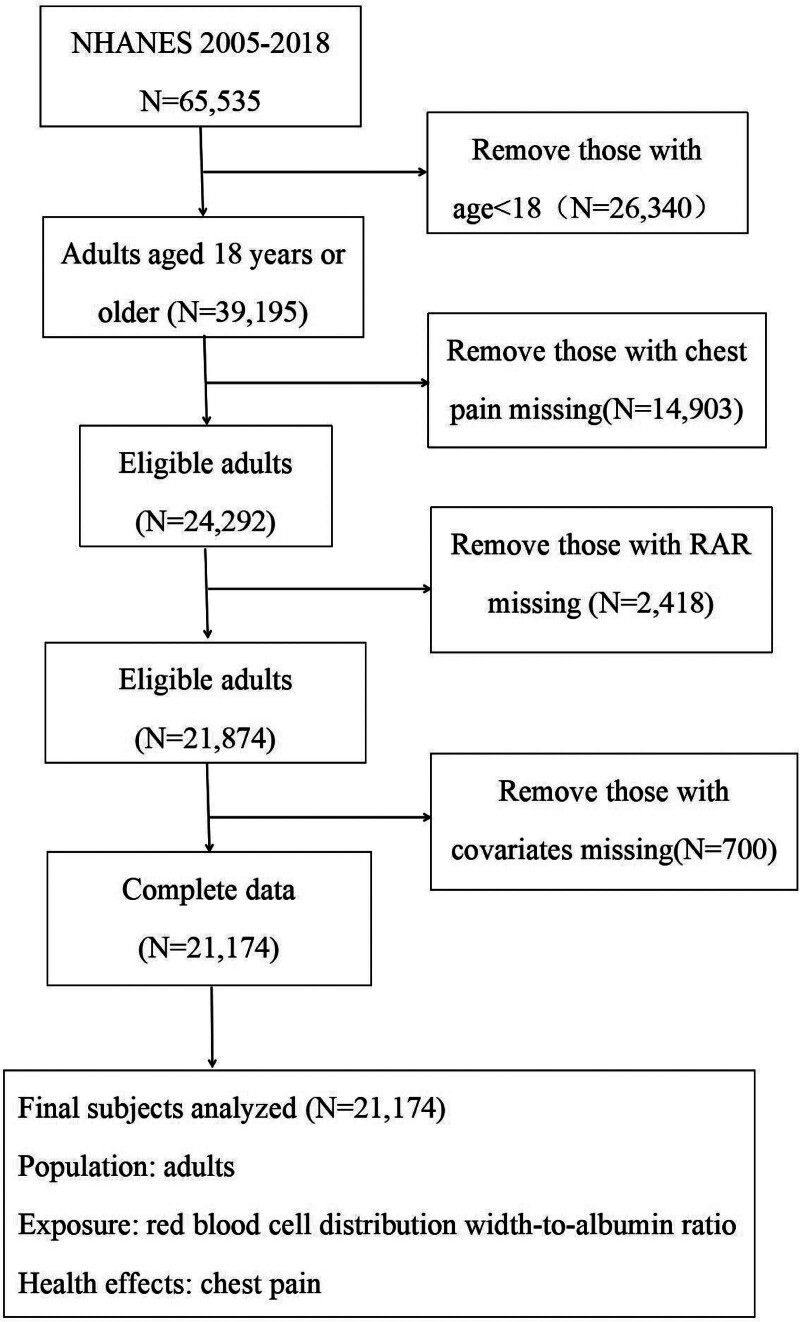
Flow diagram of the inclusion criteria and exclusion criteria. RAR = red blood cell distribution width-to-albumin ratio.

For this study, we combined data from 7 NHANES cycles (2005–2018), initially including 65,535 participants. After excluding individuals under 18 years of age (n = 26,340), those lacking chest pain data (n = 14,903), missing RAR values (n = 2418), or incomplete covariate information (n = 700), a total of 21,174 adults with complete data were retained for the final analysis (Fig. 1).

Participants with missing information on chest pain, RAR values, or key covariates were excluded from the final analysis. Listwise deletion was applied, and no imputation method was used, given the relatively low proportion of missing data and to maintain analytic transparency.

#### 2.1.1. Ethical approval declarations

Ethical approval for the NHANES protocol was obtained from the National Center for Health Statistics’ institutional review board. Written informed consent was acquired from all study participants beforehand. The current analysis utilized de-identified, open-access NHANES data, thereby exempting it from requiring further ethical clearance or consent procedures.

### 2.2. Chest pain

Chest pain was assessed using the standardized Rose Angina Questionnaire, administered by trained personnel through computer-assisted personal interviews (CAPI) conducted in participants’ homes. Participants were classified as having chest pain based on an affirmative response to the question: “Have you ever had any pain or discomfort in your chest?” This binary classification (yes/no) was applied in all subsequent statistical analyses.^[16]^

### 2.3. Measurements of RAR

RAR was calculated by dividing RDW (%) by serum albumin concentration (g/dL). RDW was obtained from peripheral blood samples using the Coulter^®^ DxH 800 analyzer, following standardized NHANES laboratory procedures conducted at Mobile Examination Centers. Serum albumin was measured using the bromocresol purple method, with the absorbance of the albumin–bromocresol purple complex recorded at 600 nm via spectrophotometry.^[17,18]^ Participants were categorized into 4 RAR quartiles based on the population distribution: Q1 (<2.91, reference), Q2 (2.91–3.13), Q3 (3.13–3.42), and Q4 (>3.42).

### 2.4. Covariates

A broad range of covariates known to influence both RAR and chest pain were included in the analysis. Demographic variables comprised age (continuous), sex (male/female), and race/ethnicity (Non-Hispanic White, Non-Hispanic Black, Mexican American, Other Hispanic, Other/Multiracial). Socioeconomic factors included education level (<high school, high school/GED, >high school), marital status (married/cohabiting, widowed/divorced/separated, never married), and the family poverty-income ratio.^[19]^ Behavioral factors included smoking status, classified as nonsmokers (<100 lifetime cigarettes) or smokers (≥100 cigarettes). Anthropometric status was assessed via body mass index (BMI), categorized as normal (<25 kg/m²), overweight (25–30 kg/m²), or obese (≥30 kg/m²). Clinical comorbidities, including hypertension, diabetes, coronary heart disease, heart failure, angina, myocardial infarction, and stroke, were identified through self-reported physician diagnoses.^[20]^

### 2.5. Statistical analyses

All statistical analyses were conducted using R software (version 4.3.0), Zstats v1.0 (www.zstats.net), and EmpowerStats (version 5.0). NHANES sampling weights were applied throughout to account for the complex multistage survey design and to ensure nationally representative estimates. Continuous variables were summarized as weighted means with standard errors, while categorical variables were presented as weighted proportions. The association between RAR and chest pain was examined using weighted multivariable logistic regression, with results reported as adjusted odds ratio (OR) and 95% confidence interval (CI). Two progressively adjusted models were constructed: Model 1 controlled for demographic variables (age, sex, and race/ethnicity), while Model 2 additionally adjusted for socioeconomic factors (education level, marital status, poverty-income ratio), behavioral factors (smoking status), and clinical conditions (BMI, hypertension, diabetes, and stroke). Potential nonlinear relationships between RAR and chest pain were explored using generalized additive models with smoothing splines; when significant nonlinearity was detected (*P* < .05), piecewise linear regression was applied to identify inflection points using maximum likelihood estimation. Subgroup analyses were conducted across categories of age (<60 vs ≥60 years), sex, race/ethnicity, marital status, BMI, RAR quartile, smoking status, hypertension, diabetes, and stroke history. Interaction effects were assessed using likelihood ratio tests, with statistical significance defined as a 2-sided *P*-value < .05.

No adjustment for multiple comparisons was performed in the subgroup analyses. Given their exploratory nature, these analyses were conducted to identify potential effect modifiers and generate hypotheses. Accordingly, findings from subgroup comparisons should be interpreted with caution.

## 3. Results

### 3.1. Characteristics of the participants

A total of 21,174 adults were included in the final analysis, among whom 5513 (26.04%) reported experiencing chest pain. Table 1 presents baseline characteristics stratified by chest pain status. The overall mean age was 59.52 ± 12.28 years, with individuals reporting chest pain being slightly older than those without (59.81 ± 12.08 vs 59.41 ± 12.35; *P* = .022). There were no significant differences in sex distribution between the 2 groups (*P* = .097). Significant racial disparities were observed: Non-Hispanic White individuals comprised a larger proportion of the chest pain group compared to those without chest pain (48.99% vs 43.97%), while Mexican Americans were underrepresented (12.21% vs 15.32%; *P* < .001). Socioeconomic differences were also evident, with the chest pain group exhibiting a lower mean poverty-income ratio (2.43 ± 1.54 vs 2.71 ± 1.56; *P* < .001). However, no significant difference was found in educational attainment between groups (*P* = .206). Marital status differed significantly, as a smaller proportion of chest pain participants were married or cohabiting (58.53% vs 64.01%; *P* < .001). Clinically, participants reporting chest pain exhibited a significantly higher cardiometabolic burden. Their mean BMI was greater compared to those without chest pain (30.32 ± 7.14 vs 29.15 ± 6.48 kg/m²; *P* < .001). The chest pain group also showed notably higher prevalence rates of diabetes (25.50% vs 18.79%), heart failure (10.07% vs 2.48%), coronary heart disease (13.95% vs 2.94%), angina (10.83% vs 1.08%), and myocardial infarction (15.24% vs 2.55%; all *P* < .001). Additionally, smoking was more common among individuals with chest pain (55.03% vs 45.64%; *P* < .001). The chest pain group exhibited a significantly higher mean RAR compared to those without chest pain (3.28 ± 0.53 vs 3.20 ± 0.47; *P* < .001). A clear dose-response trend was observed across RAR quartiles, with chest pain prevalence increasing from 21.57% in Q1 to 29.64% in Q4 (*P* < .001). This graded pattern supports the potential utility of RAR as a biomarker for chest pain risk.

**Table 1:** Baseline characteristics of participants with or without chest pain.

Characteristics	Overall (n = 21,174)	No pain (n = 15,661)	Pain (n = 5513)	*P*-value
Age	59.52 ± 12.28	59.41 ± 12.35	59.81 ± 12.08	.022
PIR	2.64 ± 1.56	2.71 ± 1.56	2.43 ± 1.54	<.001
BMI	29.45 ± 6.68	29.15 ± 6.48	30.32 ± 7.14	<.001
Gender				.097
Male	10,297 (48.63%)	7669 (48.97%)	2628 (47.67%)	
Female	10,877 (51.37%)	7992 (51.03%)	2885 (52.33%)	
Race				<.001
Mexican American	3073 (14.51%)	2400 (15.32%)	673 (12.21%)	
Other Hispanic	2051 (9.69%)	1555 (9.93%)	496 (9.00%)	
Non-Hispanic White	9587 (45.28%)	6886 (43.97%)	2701 (48.99%)	
Non-Hispanic Black	4418 (20.87%)	3180 (20.31%)	1238 (22.46%)	
Other Race	2045 (9.66%)	1640 (10.47%)	405 (7.35%)	
Education level				.206
Less than high school	5828 (27.52%)	4291 (27.40%)	1537 (27.88%)	
High school	4850 (22.91%)	3552 (22.68%)	1298 (23.54%)	
Above high school	10,496 (49.57%)	7818 (49.92%)	2678 (48.58%)	
Marital status				<.001
Married/living with partner	13,252 (62.59%)	10,025 (64.01%)	3227 (58.53%)	
Widowed/divorced/separated	6221 (29.38%)	4442 (28.36%)	1779 (32.27%)	
Never married	1701 (8.03%)	1194 (7.62%)	507 (9.20%)	
Diabetes				<.001
Yes	4348 (20.53%)	2942 (18.79%)	1406 (25.50%)	
No	16,826 (79.47%)	12,719 (81.21%)	4107 (74.50%)	
Heart failure				<.001
Yes	943 (4.45%)	388 (2.48%)	555 (10.07%)	
No	20,231 (95.55%)	15,273 (97.52%)	4958 (89.93%)	
Coronary heart disease				<.001
Yes	1229 (5.80%)	460 (2.94%)	769 (13.95%)	
No	19,945 (94.20%)	15,201 (97.06%)	4744 (86.05%)	
Angina				<.001
Yes	766 (3.62%)	169 (1.08%)	597 (10.83%)	
No	20,408 (96.38%)	15,492 (98.92%)	4916 (89.17%)	
Heart attack				<.001
Yes	1239 (5.85%)	399 (2.55%)	840 (15.24%)	
No	19,935 (94.15%)	15,262 (97.45%)	4673 (84.76%)	
Stroke				<.001
Yes	1100 (5.20%)	623 (3.98%)	477 (8.65%)	
No	20,074 (94.80%)	15,038 (96.02%)	5036 (91.35%)	
Smoking				<.001
Yes	10,181 (48.08%)	7147 (45.64%)	3034 (55.03%)	
No	10,993 (51.92%)	8514 (54.36%)	2479 (44.97%)	
RAR	3.22 ± 0.49	3.20 ± 0.47	3.28 ± 0.53	<.001
RAR group				<.001
Q1	5283 (24.95%)	4094 (26.14%)	1189 (21.57%)	
Q2	5303 (25.04%)	4040 (25.80%)	1263 (22.91%)	
Q3	5291 (24.99%)	3864 (24.67%)	1427 (25.88%)	
Q4	5297 (25.02%)	3663 (23.39%)	1634 (29.64%)	

BMI = body mass index, PIR = poverty income ratio.

### 3.2. Association of RAR with chest pain

Table 2 illustrates a robust, graded association between RAR and chest pain across all models. In the unadjusted analysis, each 1-unit increase in RAR was associated with a 38% higher odds of chest pain (OR = 1.38, 95% CI: 1.30–1.46; *P* < .0001). Individuals in the highest RAR quartile (Q4: 3.42–10.22) had 54% higher odds compared to those in the lowest quartile (Q1), with a significant trend across quartiles (OR = 1.54, 95% CI: 1.41–1.68; *P* for trend < .0001). This association remained largely unchanged after adjusting for demographic variables in Model I (age, sex, race/ethnicity), where the per-unit OR was 1.38 (95% CI: 1.30–1.47) and Q4 vs Q1 OR was 1.55 (95% CI: 1.41–1.69; both *P* < .0001). In the fully adjusted Model II, which additionally accounted for socioeconomic status (education, marital status, and poverty-income ratio), behavioral factors (smoking), and clinical comorbidities (BMI, hypertension, diabetes, and stroke), the association remained significant though attenuated. Each unit increase in RAR was linked to 19% higher odds of chest pain (OR = 1.19, 95% CI: 1.12–1.27; *P* < .0001), and participants in Q4 continued to show elevated risk compared to Q1 (OR = 1.26, 95% CI: 1.15–1.39; *P* < .0001). The third quartile (Q3: 3.13–3.42) also showed a significantly increased risk (OR = 1.16, 95% CI: 1.06–1.27; *P* = .0019), while the second quartile (Q2: 2.91–3.13) did not reach statistical significance (OR = 1.04, 95% CI: 0.94–1.14; *P* = .4522). A significant linear trend across quartiles persisted in the fully adjusted model (*P* for trend = .0001).

**Table 2:** Association between RAR and chest pain.

Exposure	Non-adjusted model	Model I	Model II
RAR	1.38 (1.30, 1.46) < 0.0001	1.38 (1.30, 1.47) < 0.0001	1.19 (1.12, 1.27) < 0.0001
Q1 (2.19–2.91)	Ref	Ref	Ref
Q2 (2.91–3.13)	1.08 (0.98, 1.18) 0.1101	1.09 (0.99, 1.19) 0.0789	1.04 (0.94, 1.14) 0.4522
Q3 (3.13–3.42)	1.27 (1.16, 1.39) < 0.0001	1.28 (1.17, 1.40) < 0.0001	1.16 (1.06, 1.27) 0.0019
Q4 (3.42–10.22)	1.54 (1.41, 1.68) < 0.0001	1.55 (1.41, 1.69) < 0.0001	1.26 (1.15, 1.39) < 0.0001
*P* for trend	<.0001	<.0001	.0001

Model 1 adjust for: sex, age, race; Model 2 adjust for: sex, age, race, education level, marital status, PIR, BMI, hypertension, diabetes, stroke, and smoking.

BMI = body mass index, PIR = poverty income ratio, RAR = red blood cell distribution width-to-albumin ratio.

#### 3.2.1. Restricted cubic spline regression analysis between RAR and chest pain

**Figure 2.**
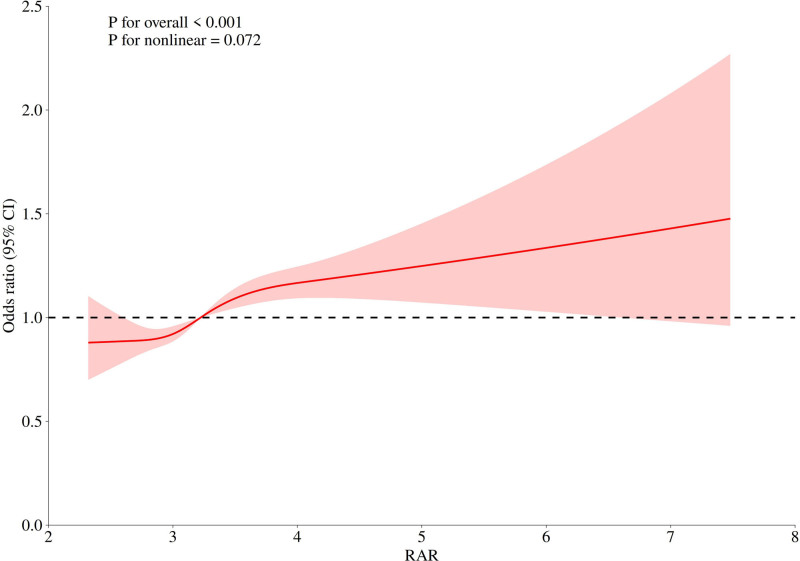
The smoothed curve-fit plot of the dose-response relationship between RAR and chest pain. CI = confidence interval, RAR = red blood cell distribution width-to-albumin ratio.

Figure 2 illustrates a significant nonlinear relationship between RAR and chest pain risk (*P* for overall < .001; *P* for nonlinearity = .072). The restricted cubic spline curve shows a sharp increase in chest pain risk at lower RAR levels (approximately 2.0–3.5), followed by a plateau beyond a defined threshold. Table 3 supports this threshold effect, with 2-piecewise logistic regression identifying an inflection point at RAR = 3.59 (*P* for log-likelihood ratio test = .045). Below this threshold (RAR < 3.59), each unit increase in RAR was associated with a 32% higher odds of chest pain (OR = 1.32, 95% CI: 1.17–1.49; *P* < .0001). In contrast, above the threshold (RAR ≥ 3.59), the association was no longer statistically significant (OR = 1.09, 95% CI: 0.97–1.22; *P* = .1417). This nonlinear pattern remained robust after adjustment for demographic, socioeconomic, and clinical covariates.

**Table 3:** Nonlinearity addressing of RAR and chest pain.

Chest pain	OR (95% CI), *P*-value
RAR	
Fitting model by standard logistic regression	1.19 (1.12, 1.27), <.0001
Fitting model by 2-piecewise logistic regression	
Inflection point	3.59
<3.59	1.32 (1.17, 1.49), <.0001
>3.59	1.09 (0.97, 1.22), .1417
*P* for log likely ratio test	.045

Adjusted for all covariates presented in Table 2.

CI = confidence interval, OR = odds ratio, RAR = red blood cell distribution width-to-albumin ratio.

### 3.3. Subgroup analyses

**Figure 3.**
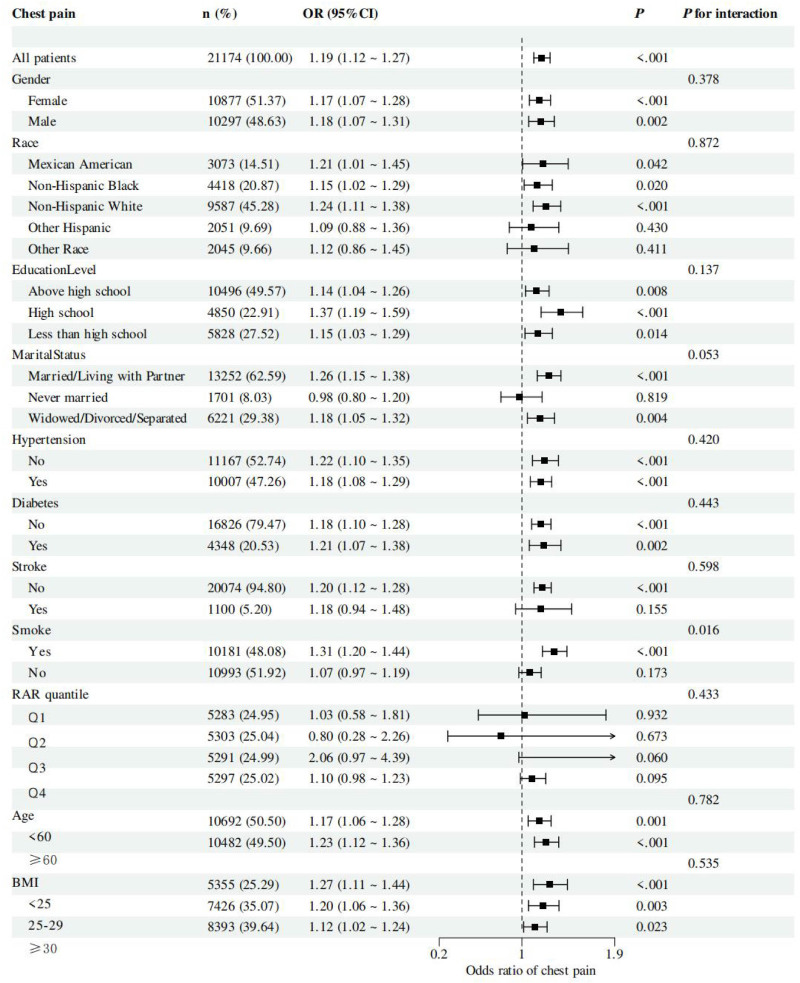
Subgroup analysis for the association between RAR and chest pain. BMI = body mass index, CI = confidence interval, OR = odds ratio, RAR = red blood cell distribution width-to-albumin ratio.

Figure 3 presents subgroup analyses, revealing notable variation in the association between RAR and chest pain across population strata. A significant interaction was observed with smoking status (*P* for interaction = .016), where current smokers showed a stronger association (OR = 1.31, 95% CI: 1.20–1.44; *P* < .001) compared to nonsmokers (OR = 1.07, 95% CI: 0.97–1.19; *P* = .173), suggesting that smoking may amplify RAR-related inflammatory and oxidative mechanisms. The association remained consistent across sex and age groups. Similar effect sizes were observed in males and females, with no significant interaction (*P* = .378). Associations were also evident in both younger and older adults (*P* for interaction = .423). By race/ethnicity, the association was strongest in Non-Hispanic Whites and Mexican Americans, though no significant interaction was found (*P* = .872). Some subgroups, including Other Hispanic and Other Race, did not show statistically significant associations. The relationship between RAR and chest pain was not modified by hypertension (*P* = .420) or diabetes (*P* = .443), with comparable effect sizes across groups. In BMI-stratified analyses (*P* for interaction = .535), the strongest association was observed in normal-weight individuals (OR = 1.77, 95% CI: 1.11–1.46). Overall, these findings underscore the robustness of the RAR – chest pain association across most subgroups, particularly among smokers, while also highlighting variability in specific populations that merits further investigation.

## 4. Discussion

In this nationally representative cross-sectional study using NHANES 2005–2018 data, we found a significant positive association between higher RAR levels and chest pain prevalence. A nonlinear relationship was observed, with a threshold effect at RAR = 3.59. Below this point, each unit increase in RAR was associated with 32% higher odds of chest pain (OR = 1.32, 95% CI: 1.17–1.49), whereas the association diminished beyond the threshold. Subgroup analyses revealed a particularly strong effect among current smokers (*P* for interaction = .016), with consistent associations across age and racial/ethnic groups, although effect sizes varied. These findings support the utility of RAR as a composite biomarker reflecting both inflammatory and nutritional status in assessing chest pain risk. The observed threshold may have clinical relevance in identifying individuals, especially smokers, who may benefit from interventions targeting systemic inflammation and nutritional deficiencies.

To our knowledge, this is the first large-scale epidemiological study to investigate the association between RAR and chest pain in a nationally representative population. Several clinical studies have highlighted the potential role of RAR and its components in cardiovascular conditions including chest pain and arrhythmia.^[21]^ For example, Li and Song^[22]^ found that higher RAR values were independently associated with an increased risk of atrial fibrillation among patients undergoing coronary angiography. A retrospective cohort study of elderly patients in China showed that elevated RAR was significantly correlated with 28-day cardiovascular mortality among patients with atrial fibrillation.^[10]^ Additionally, Eyiol and Ertekin^[14]^ reported that a lower hemoglobin-to-RDW ratio was predictive of greater stroke severity and in-hospital mortality, suggesting RDW’s strong prognostic value in acute vascular presentations. Our findings are broadly consistent with these studies, showing that elevated RAR is positively associated with chest pain prevalence in US adults, based on NHANES data. Together, these findings reinforce the inflammatory-nutritional basis of RAR as a biomarker of cardiovascular stress and subclinical ischemia.

In this comprehensive analysis, we utilized multivariable logistic regression, restricted cubic spline modeling, and detailed subgroup analyses to explore the relationship between RAR and chest pain. Our findings indicate that elevated RAR levels – particularly those exceeding 3.59 – are significantly associated with increased chest pain prevalence, with the strongest effects observed among smokers and older adults. The nonlinear pattern and threshold at RAR = 3.59 suggest a potential saturation effect, where inflammatory and nutritional dysregulation may reach a plateau in their impact on chest pain risk.^[23]^ Although the association was attenuated after adjusting for demographic, behavioral, and clinical factors, it remained statistically significant (OR = 1.26 for Q4 vs Q1), supporting RAR’s potential as an independent risk indicator.

Emerging evidence increasingly supports the view that chest pain, particularly of cardiovascular origin, is intricately linked to systemic inflammation and metabolic dysregulation.^[24]^ Elevated RDW, a key component of RAR, reflects anisocytosis driven by oxidative stress, chronic inflammation, and impaired erythropoiesis, all of which can promote endothelial dysfunction and contribute to atherosclerotic plaque instability – core mechanisms in ischemic chest pain development.^[25,26]^ Simultaneously, hypoalbuminemia, the other component of RAR, is not merely a marker of malnutrition but also a negative acute-phase reactant, often suppressed in inflammatory states.^[10,27]^ Low albumin levels have been associated with reduced antioxidant capacity, impaired vascular integrity, and increased inflammatory cytokine activity, such as interleukin-6 and tumor necrosis factor-alpha.^[27–29]^ Our findings support the hypothesis that RAR reflects cumulative physiological stress arising from both inflammatory activation and nutritional deficits, which may exacerbate chest pain via multiple biological pathways.^[24]^ These include impaired oxygen delivery, microvascular dysfunction, and heightened vascular reactivity.^[30]^ The observed threshold effect at RAR = 3.59 may indicate a saturation point beyond which the inflammatory-nutritional burden exerts maximal clinical impact. Additionally, smoking – a significant modifier in our subgroup analysis – may potentiate these mechanisms by intensifying oxidative stress and impairing endothelial repair capacity.^[31]^ It is also possible that nutritional inadequacies associated with low serum albumin contribute to suboptimal myocardial energy metabolism, thereby amplifying pain perception during ischemic episodes.^[32]^ The relationship between RAR and chest pain may also be bidirectional. Repeated ischemic injury can elevate systemic inflammation, further suppressing albumin synthesis and driving RDW elevation, thereby creating a feedback loop that sustains or aggravates chest pain symptoms.^[33]^ Beyond cardiovascular causes, inflammation and oxidative stress – reflected by high RAR – are increasingly recognized in musculoskeletal and visceral pain pathways, suggesting that RAR may be relevant to a broader spectrum of chest pain etiologies.^[34,35]^ However, the precise biological pathways mediating these associations remain to be fully elucidated and warrant further experimental and longitudinal investigation.

These findings hold particular relevance for high-risk populations, including smokers and individuals with cardiometabolic conditions, in whom elevated RAR may indicate increased inflammatory burden. Future longitudinal and interventional studies are warranted to establish causality and assess the clinical utility of RAR in guiding personalized strategies for chest pain management.

### 4.1. Limitations

This study has several limitations. First, although we adjusted for a wide range of demographic, socioeconomic, behavioral, and clinical covariates, the possibility of residual confounding due to unmeasured or unknown variables cannot be excluded. Sencond, given the cross-sectional nature of NHANES, causality cannot be inferred. The observed associations between RAR and chest pain reflect correlations at a single time point and do not establish temporal directionality. Prospective studies are needed to confirm these findings. Third, chest pain was assessed using the Rose Angina Questionnaire, a self-reported tool. While this questionnaire is widely validated and used in epidemiological research, it is still subject to potential misclassification bias due to recall inaccuracies or subjective interpretation by participants. Such non-differential misclassification may have biased our estimates toward the null, potentially underestimating the true strength of association. Moreover, the findings may not be generalizable to non-US populations, as RDW and albumin distributions may vary across ethnic and regional groups. Finally, although we conducted multiple subgroup analyses to explore potential effect modification, we did not apply statistical corrections for multiple comparisons. As such, the possibility of type I error (false positives) cannot be excluded. These subgroup findings should therefore be viewed as exploratory and interpreted with caution.

## 5. Conclusion

This study identifies RAR as an independent predictor of chest pain, with a significant nonlinear relationship and a threshold effect observed at RAR = 3.59. The association was most pronounced among smokers (OR = 1.31), underscoring its potential utility for risk stratification in pro-inflammatory populations. Given its accessibility and integrative reflection of inflammatory and nutritional status, RAR may serve as a valuable biomarker in clinical evaluation – particularly in individuals with cardiometabolic risk. Prospective studies are warranted to confirm these findings and explore RAR’s role in guiding preventive or therapeutic strategies.

## Acknowledgments

We extend our heartfelt appreciation to the staff of the National Center for Health Statistics, under the Centers for Disease Control. Their diligent work in designing, collecting, and collating NHANES data, as well as creating the public database, is highly commendable and has significantly contributed to public health research.

## Author contributions

**Methodology:** Minmin Zhu.

**Funding acquisition:** Dongxiao Huang.

**Supervision:** Hao Xi.

**Writing – original draft:** Minmin Zhu.

**Writing – review & editing:** Hao Xi.
